# A Nationwide Danish Comparative Effectiveness Study of GLP‐1 RA, SGLT2i and DPP‐4i Treatment on Risk of Stroke, Myocardial Infarction and Mortality in Type 2 Diabetes

**DOI:** 10.1002/edm2.70165

**Published:** 2026-01-24

**Authors:** Sidsel Hastrup, Jakob N. Hedegaard, Grethe Andersen, Merete Osler, Jørgen Rungby, Søren Paaske Johnsen

**Affiliations:** ^1^ Department of Neurology, Danish Stroke Center Aarhus University Hospital Aarhus Denmark; ^2^ Department of Clinical Medicine, Danish Center for Health Services Research Aalborg University Aalborg Denmark; ^3^ Department of Clinical Medicine University of Aarhus Aarhus Denmark; ^4^ Center for Clinical Research and Prevention Bispebjerg and Frederiksberg Hospitals Frederiksberg Denmark; ^5^ Section of Epidemiology, Department of Public Health University of Copenhagen Copenhagen Denmark; ^6^ Steno Diabetes Center Copenhagen Herlev Denmark; ^7^ Department of Clinical Medicine University of Copenhagen Copenhagen Denmark

**Keywords:** DPP‐4i, GLP‐1 RA, SGLT2i, stroke, type 2 diabetes

## Abstract

**Aims:**

Cardiovascular outcome trials have demonstrated that glucagon‐like peptide‐1 receptor agonists (GLP‐1 RA) and sodium–glucose cotransporter 2 inhibitors (SGLT2i) reduce the risk of major adverse cardiovascular events, whereas dipeptidyl peptidase‐4 inhibitors (DPP‐4i) have not shown cardiovascular benefits. We aimed to compare the effectiveness in routine clinical settings of incident use of either GLP‐1 RA, SGLT2i or DPP‐4i among type 2 diabetes on the stroke risk and as secondary outcomes myocardial infarction and all‐cause mortality.

**Methods:**

A nationwide population‐based cohort study consisted of persons with type 2 diabetes who were new users of a GLP‐1 RA, SGLT2i or DPP‐4i and without prior stroke from 2014 to 2020 in Denmark using an active comparator design. They were followed from initiation of medication up to a maximum of 2 years for incident outcomes. Estimates were adjusted for age, sex, calendar year of initiation, socio‐economic factors, medication and co‐morbidity.

**Results:**

The study included 19,999 new users of a GLP‐1 RA; 24,702 of a SGLT2i and 41,943 of a DPP‐4i. The new users of GLP‐1 RA had a lower incidence of stroke when compared to new users of DPP‐4i, adjusted hazard rate ratios (aHRR): 0.69 95% confidence interval (0.53–0.91). There was no significant difference in stroke incidence between the new users of SGLT2i versus DPP4‐4i and SGLT2i versus GLP‐1 RA: aHRR 0.80 (0.64–1.01) and 1.17 (0.87–1.57). The new users of GLP‐1 RA and SGLT2i had lower risk of mortality in comparison with new users of DPP‐4i. The risk of myocardial infarction was not significantly different between the compared groups.

**Conclusions:**

New users of GLP‐1 RA with type 2 diabetes had a lower risk of first stroke and new users of GLP‐1 RA and SGLT2i had lower mortality. These data could help guide the choice of glucose‐lowering medications in persons with type 2 diabetes.

## Graphical abstract

New users of GLP‐1 RA in type 2 diabetes had a lower risk of stroke and new users of GLP‐1 RA and SGLT2i in type 2 diabetes had a lower risk of mortality in comparison with new users of DPP‐4i in routine clinical settings. There were no differences in myocardial infarction.

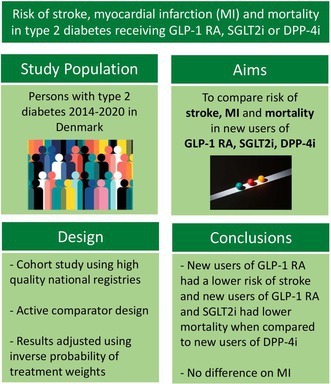

## 1 Introduction

Type 2 diabetes is a major modifiable risk factor for stroke. Thus, the risk of a stroke is approximately twofold higher in persons with type 2 diabetes compared with those without [1]. The occurrence of stroke in type 2 diabetes also results in high costs and healthcare resource utilisation compared with type 2 diabetes alone and compared to various other cardiovascular events or conditions [2, 3]. Furthermore, stroke patients with co‐morbid type 2 diabetes have an adverse prognosis with increased risks of recurrent stroke, mortality, readmissions and poorer cognitive outcome [4, 5, 6, 7]. Despite this, there is a paucity of available treatments that specifically target the risk of stroke in persons with type 2 diabetes [8]. Overall, stroke in type 2 diabetes represents a key area of unmet need for both patients and healthcare systems.

A series of cardiovascular outcome trials have created robust clinical evidence of cardiovascular benefits of glucagon‐like peptide‐1 receptor agonists (GLP‐1 RA) and sodium–glucose cotransporter 2 inhibitors (SGLT2i) in persons with type 2 diabetes and high cardiovascular risk or established atherosclerotic cardiovascular disease with a reduction in major adverse cardiovascular events [9]. Two cardiovascular outcome trials using GLP‐1 RA also demonstrated a significant reduction in the risk of non‐fatal stroke [10, 11] and a subsequent meta‐analysis of all the cardiovascular outcome trials of GLP‐1 RAs found a reduction in the risk of fatal or non‐fatal stroke [12]. As SGLT2i with an indication to treatment of type 2 diabetes did not show a reduction in stroke and dipeptidyl peptidase‐4 inhibitors (DPP‐4i) have not shown cardiovascular benefits in randomised clinical [13], the recent stroke‐ and diabetes guidelines recommend the use of GLP‐1 RA in type 2 diabetes patients with a history of ischemic stroke or transient ischemic attack [9, 14, 15]. However, as the randomised trials individually only evaluated one glucose lowering medication versus placebo, a head‐to‐head comparative randomised trial of the newer drug classes (SGLT2i vs. GLP‐1 RA vs. DPP‐4i) on risk of major adverse cardiovascular events as well as stroke is not available. Real world evidence studies comparing the newer anti‐diabetic drug classes on the risk of stroke in broader populations are also sparse.

In this study, we aimed to compare the effectiveness of incident use of either a GLP‐1 RA, SGLT2i or DPP‐4i among persons with type 2 diabetes and without prior stroke on the risk of stroke (ischemic and hemorrhagic) and as secondary outcomes on the risk of myocardial infarction and all‐cause mortality.

## 2 Materials and Methods

### 2.1 Study Design

The study was a nationwide, register‐based cohort study utilising the New User/Active Comparator design [16] to estimate the effects of being continuously exposed to either GLP‐1 RA, SGLT2i or DPP‐4i (newer glucose‐lowering medications) in a population composed of persons with type 2 diabetes in Denmark. To do so, prescription use of newer glucose‐lowering medications in persons with type 2 diabetes in Denmark was tracked over time, allowing for the definition of subpopulations of type 2 diabetes who were exposed to either of the glucose‐lowering medications and none of the competing medications. These persons were compared in a time‐to‐event setting, comparing rates of occurrence of the primary outcome: Stroke (ischemic and hemorrhagic). The secondary outcomes were myocardial infarction and all‐cause mortality.

### 2.2 Data Sources

The study is based upon high quality Danish healthcare registries linked by a 10‐digit social registry number, which is provided to and is unique for every Danish citizen. We used information from the Danish Civil Registration System considered to have a high validity [17], which contains information on vital status including date of birth, migration, emigration and death, sex and other demographic information; the Danish National Patient Registry considered a valuable tool for epidemiological research and overall a high validity and completeness although with variation in diseases and treatments [18], which contains information on hospitalizations and procedures undertaken in the Danish non‐psychiatric hospitals; the Danish National Prescription Registry [19], which includes a history of medicine prescriptions. Data in the Danish National Prescription Registry are considered complete and valid as from 1995. The use of bare codes throughout the dispensing process at Danish pharmacies minimises the risk of data entry errors; the Register of Laboratory Results for Research with complete nationwide coverage since 2015 and with a high data quality [20], containing results from biochemical analyses and various information obtained from Statistics Denmark [21], including those relating to income, educational level and immigration status.

### 2.3 Study Setting and Participants

**FIGURE 1:**
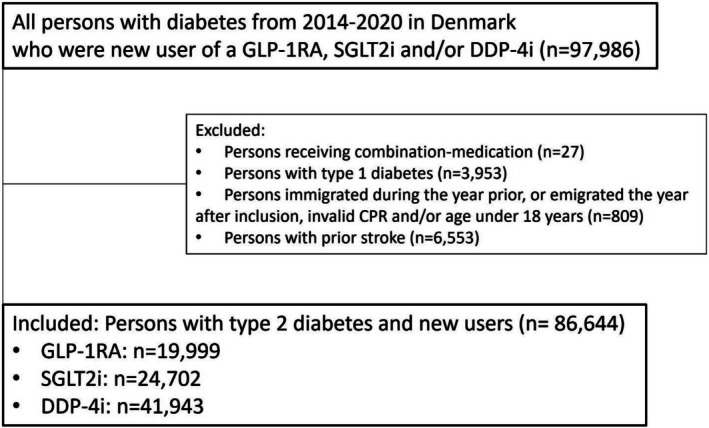
Flowchart of the study cohort. Civil Personal Registration number (CPR).

The study population was based on the entire Danish population, and consisted of persons with type 2 diabetes who were new and active users of one, and only one, of the drug classes: GLP‐1 RA; SGLT2i or DPP‐4i, who (a) started medicine use between 2014 and 2020 (b) were at least 18 years (c) had information on death in the Danish Civil Registration System, and (d) had no prior strokes (but could have a history of another prior cardiovascular including myocardial infarction) at the time of medicine initiation. We refer to Figure 1 for detailed information on the inclusion process in the study. See Appendix S1 for a list of the ATC‐codes used for defining the three drug categories. As the risk of stroke and recurrent myocardial infarction, respectively, is high [22], additional sensitivity analyses of the outcomes excluding persons with prior TIA and myocardial infarction, respectively, were performed.

A person was defined as having type 2 diabetes based on the medical history obtained from the Danish registries. Specifically, we obtained hospital diagnoses and histories of prescription medicine, and defined a person with either type 1 or type 2 diabetes if the person had received GLP‐1 RA; SGLT2i or DPP‐4i with antidiabetic indication. Individuals with polycystic ovary syndrome, gestational diabetes and obesity/no diabetes indications were excluded. We classified diabetes as type 1 or type 2 according to age of first use of insulin and insulin analogues, and the ICD‐10 diagnosis codes associated with hospital contacts. See Appendix S1 for details. The study population was restricted to individuals with type 2 diabetes.

### 2.4 Variables

In this study we defined a three‐levelled exposure corresponding to the type of newer glucose‐lowering medication used, three clinical outcomes, various covariates used for adjusting for potential confounding, and a range of additional variables used to characterise the population.

#### 2.4.1 Exposure

For each included person, we defined periods of continuous use of one of GLP‐1 RA, SGLT2i or DPP‐4i via daily‐defined doses.

Patients were defined as at risk while continuous use was present, and censored if there was cessation of medicine use or changed to one of the ‘conflicting medicines’. A grace period of 90 days and a wash‐out period of 30 days was used. For patients with multiple separate periods of medicine use, only the first was included.

#### 2.4.2 Outcomes

The primary outcome was stroke (ICD‐10: I61, I63 and I64) and the secondary outcomes were myocardial infarction (ICD‐10: I21 and I22) and all‐cause mortality.

#### 2.4.3 Covariates

We characterised our patients according to age, sex assigned at birth, comorbidity registered prior to exposure (up to 15 years prior), socio‐economic factors, laboratory results (HbA1c, cholesterol (total and LDL) and eGFR) closest in time measurement to date of exposure among measurements taken during the year prior, medicine prior to exposure (based on prescriptions in the year prior). Age, sex assigned at birth, calendar year of medicine initiation, ethnicity, habitation status, income, educational level, duration of diabetes, hypertension, atrial fibrillation and Charlson Comorbidity Index (CCI) [23], medicine prior to exposure (statins, antiplatelets, anticoagulation (Vitamin K and DOAC), antihypertensive drugs) and chronic complications (e.g., nephropathy, neuropathy and angiopathy; please see Appendix S1 for further details on ICD‐10 codes) of diabetes were considered as potential confounding factors as they are known important risk factors of stroke [24] and were controlled for. Laboratory results covariates were only used in a descriptive manner. See Appendix S1 for further details on covariates and their definition. Furthermore, information about the exposure such as time at risk, year of initiation, percentage of risk periods ending in a change to a conflicting medicine, percentage with a risk time less than 30 days and similar were recorded and used descriptively. All confidence intervals (CI) were calculated with a 95% confidence level.

### 2.5 Statistical Analysis

Summary statistics were reported for the covariates in Table 1. The clinical outcomes were analysed in a time‐to‐event analysis using Aalen‐Johansen to derive cumulative incidences reported in Table 2, and Cox regression to obtain cause‐specific hazard rate ratios (HRR) reported in Table 3. In the analysis of stroke, death was considered a competing risk, and myocardial infarction was not. The analysis of myocardial infarction was conducted similarly. Patients were counted as at risk from the date of the first prescription, and they were censored if they were alive and event free at the end of their exposure. Furthermore, administrative censoring at 730 days after drug initiation was also used when outcomes were compared. Cumulative incidences of outcomes were computed after 365 and 730 days in order to ensure standardised and comparable follow‐up time as well as detailed insights into the development of incidences of outcomes during follow‐up. Crude and adjusted HRRs were reported. Adjustment for confounding was achieved using inverse probability of treatment weights obtained from one of three logistic propensity models (logistic regression) corresponding to each of the three comparisons, GLP‐1 RA relative to DPP‐4i, SGLT2i relative to DPP‐4i and SGLT2i relative to GLP‐1 RA.

**TABLE 1:** Baseline characteristics of study cohort.

Type 2 diabetes and new users of	GLP1‐RA	SGLT2i	DPP‐4i
Users, *n*	19,999	24,702	41,943
Year of initiation, % (*n*)			
2014	6.9 (1370)	2.1 (517)	15.8 (6617)
2015	8.1 (1617)	3.5 (871)	17.0 (7140)
2016	10.4 (2080)	7.9 (1958)	17.5 (7335)
2017	11.5 (2305)	12.8 (3164)	16.3 (6832)
2018	16.2 (3233)	18.7 (4619)	14.1 (5914)
2019	17.4 (3478)	24.0 (5930)	11.0 (4625)
2020	29.6 (5916)	30.9 (7643)	8.3 (3480)
Age, Median (IQR)	56.3 (47.2–66.3)	61.9 (53.4–70.4)	65.8 (55.8–74.4)
Male, % (*n*)	48.4 (9673)	63.2 (15,620)	59.8 (25,068)
Immigrant or descendant of immigrants, % (*n*)	11.3 (2255)	16.2 (4012)	15.4 (6469)
Habitation/lives alone, % (*n*)	30.9 (6153)	32.1 (7926)	35.8 (14,963)
Educational level, % (*n*)			
Low	31.4 (5903)	36.1 (8444)	40.0 (14,602)
Medium	48.7 (9174)	48.9 (11,435)	46.3 (16,902)
High	19.9 (3743)	15.0 (3495)	13.6 (4978)
Income > median income % (*n*)	53.5 (9816)	47.3 (11,039)	40.6 (15,955)
Duration of diabetes % (*n*)			
0–2 years	45.1 (9013)	30.7 (7577)	26.7 (11,216)
2–5 years	16.3 (3265)	22.4 (5540)	23.0 (9637)
5–10 years	19.2 (3843)	27.0 (6659)	28.7 (12,049)
> 10 years	19.4 (3878)	19.9 (4926)	21.6 (9041)
Charlson Comorbidity Index % (*n*)			
CCI = 0	76.8 (15,351)	74.0 (18,288)	67.5 (28,314)
CCI = 1	9.9 (1971)	12.1 (2994)	11.7 (4913)
CCI ≥ 2	13.4 (2677)	13.8 (3420)	20.8 (8716)
Atrial fibrillation, % (*n*)	6.6 (1320)	8.9 (2197)	10.8 (4525)
Hypertension, % (*n*)	65.5 (13,109)	74.6 (18,422)	76.6 (32,121)
Diabetes with chronic complications, % (*n*)	16.5 (3308)	14.8 (3645)	16.7 (7022)
Peripheral vascular disease, % (*n*)	4.1 (826)	4.9 (1208)	6.1 (2558)
Prior myocardial infarction, % (*n*)	5.0 (992)	8.2 (2026)	6.6 (2783)
Prior ASCVD, % (*n*)	20.2 (4043)	23.7 (5853)	24.7 (10,347)
Prior PAD, % (*n*)	6.4 (1282)	6.1 (1506)	7.2 (3033)
Prior TIA, % (*n*)	13.1 (2612)	10.4 (2571)	8.0 (3367)
Medical obesity, % (*n*)	31.8 (6356)	17.8 (4401)	14.7 (6161)
Biguanides, % (*n*)	65.8 (13,155)	89.7 (22,160)	87.5 (36,701)
Sulfonylureas, % (*n*)	10.2 (2035)	12.9 (3183)	18.7 (7856)
Glitazones, % (*n*)	0.0 (9)	0.0 (7)	0.1 (24)
Insulin and analogues, % (*n*)	19.1 (3818)	10.7 (2632)	5.9 (2477)
Meglitinides, % (*n*)	0.2 (42)	0.3 (62)	0.4 (165)
Alfa‐glucosidase inhibitors, % (*n*)	0.0 (8)	*	0.0 (13)
Statins, % (*n*)	51.7 (10,341)	67.5 (16,684)	66.8 (28,003)
Antiplatelet drug, all, % (*n*)	21.5 (4299)	27.4 (6759)	29.8 (12,494)
Acetyl Salicylic Acid, % (*n*)	19.6 (3923)	24.7 (6091)	27.3 (11,445)
Anticoagulation (Vitamin K), % (*n*)	2.9 (587)	3.3 (804)	5.8 (2449)
Anticoagulation (DOAC), % (*n*)	5.0 (995)	6.7 (1657)	5.9 (2458)
Angiotensin, % (*n*)	51.7 (10,347)	61.7 (15,249)	61.0 (25,589)
Antihypertensive drugs, all, % (*n*)	64.2 (12,848)	73.2 (18,090)	75.2 (31,552)
Cholesterol‐total (mmol/L), median (IQR)	4.4 (3.7–5.2)	4.10 (3.5–5.0)	4.2 (3.6–5.0)
Missing cholesterol value %	35.5	29.9	33.3
LDL‐cholesterol (mmol/L), median (IQR)	2.2 (1.6–3.0)	2.0 (1.5–2.7)	2.0 (1.5–2.7)
Missing LDL‐cholesterol value % (*n*)	38.5	33.3	36.9
HbA1c (mmol/mol), median (IQR)	58.0 (45.0–73.0)	62.0 (55.0–74.0)	62.0 (55.0–73.0)
Missing HbA1c value % (*n*)	32.7	27.7	30.6
eGRF(mL/min/1.73 m^2^), % (*n*)			
< 45	3.2 (639)	1.6 (406)	9.8 (4101)
45–60	4.5 (901)	4.7 (1151)	7.2 (3031)
60–90	23.4 (4676)	28.5 (7052)	25.1 (10,517)
> 90	38.0 (7593)	37.6 (9291)	27.5 (11,537)
Missing eGFR value % (*n*)	31.0	27.5	30.4

**TABLE 2:** Cumulative risk (% and 95% confidence intervals) of stroke, myocardial infarctions and all‐cause mortality after 365 and 730 days.

Drug class	Time window (days)	Population (*n*)	Strokes	Myocardial infarction (*n*)	All‐cause mortality (*n*)
GLP‐1 RA		19,999			
	365		0.4 (0.3–0.6)	0.5 (0.4–0.6)	0.7 (0.6–0.9)
	730		0.9 (0.7–1.1)	0.9 (0.8–1.2)	1.5 (1.3–1.7)
SGLT2i		24,702			
	365		0.6 (0.5–0.7)	0.8 (0.6–0.9)	0.9 (0.8–1.1)
	730		1.1 (0.9–1.3)	1.3 (1.1–1.5)	1.5 (1.3–1.7)
DPP‐4i		41,943			
	365		0.9 (0.8–1.0)	0.7 (0.7–0.8)	3.8 (3.6–4.0)
	730		1.6 (1.5–1.8)	1.3 (1.1–1.4)	6.4 (6.1–6.7)

**TABLE 3:** Comparison of new users of GLP‐1 RA, SGLT2i and DPP‐4i on stroke, myocardial infarction and all‐cause mortality.

	Population (*n*)	Stroke (*n*)	Stroke (HRR)	MI (*n*)	MI (HRR)	Mortality (*n*)	Mortality (HRR)
GLP1‐RA/DPP‐4i							
Crude	61,942	551	0.50 (0.40–0.62)	465	0.68 (0.54–0.84)	1985	0.21 (0.18–0.24)
Adjusted^a^	40,631	319	0.69 (0.53–0.91)	290	1.00 (0.76–1.32)	834	0.44 (0.36–0.53)
SGLT2i/DPP‐4i							
Crude	66,645	600	0.63 (0.52–0.76)	560	1.07 (0.90–1.27)	2042	0.23 (0.20–0.27)
Adjusted^a^	43,810	370	0.80 (0.64–1.01)	337	1.19 (0.94–1.49)	1027	0.40 (0.34–0.48)
SGLT2i/GLP‐1 RA							
Crude	44,701	249	1.26 (0.97–1.62)	309	1.59 (1.26–2.01)	389	1.13 (0.92–1.38)
Adjusted^a^	34,448	202	1.17 (0.87–1.57)	556	1.31 (1.00–1.70)	315	0.88 (0.69–1.11)

^a^ Adjusted for age, sex, calendar year of initiation, migrant status, co‐habitation status, income, education, duration of diabetes, hypertension, atrial fibrillation, Charlson Comorbidity Index (CCI), prior medication with statins, antiplatelets, anticoagulation (vitamin K and DOAC), antihypertensive drugs and diabetes‐related chronic complications.

The three propensity models were of the same form and included age as a continuous predictor. The remaining covariates were included as factor variables. The CCI was grouped into three scores: a score of zero, a score of one and a score of two or greater.

The propensity scores were used to calculate weights used to estimate average treatment effects. Trimming of the populations was performed by removing patients with propensities lower or larger than the 0.05 and 0.95 percentiles respectively, and the remaining patients were reweighted using the propensity models of the same form as the ones used to calculate the initial weights. In Appendix S1, we summarise the confounding covariates prior to and after weighting and trimming (Balance diagnostics).

Missingness in data was handled using multiple imputation with chained equations and predictive mean matching to obtain 10 completed datasets. Matching was based on the 10 nearest neighbours and results were aggregated according to Rubin's rule.

All analyses were performed using Stata 17.0, Stata Corp LLC.

### 2.6 Ethics Statement

Under Danish law, registry‐based studies require no ethical approval or patient consent. The study was approved by the Danish Data Protection Agency (P‐2020‐88) and the Danish Clinical Registries.

## 3 Results

The study included: 19,999 new users of GLP‐1 RA; 24,702 of SGLT2i; and 41,943 of DPP‐4i. The median risk times were: GLP‐1 RA was 363 (161–770) days, SGLT2i 355 (134–707) days and DPP‐4i 475 (163–1051) days.

Baseline characteristics of new users of GLP‐1 RA, SGLT2i and DPP‐4i (Table 1) showed that there were some numerical differences in year of initiation, age, sex, socio‐economic factors, duration of diabetes, use of other medications and co‐morbidity across the groups. There were also differences in HbA1c and eGFR between the groups.

Table 2 shows the cumulative incidences of the three outcomes. In the time window of 730 days there were 100 strokes among the new users of GLP‐1 RA corresponding to a cumulative incidence of 0.9% 95% CI (0.7–1.1); 149 strokes among SGLT2i users corresponding to a cumulative incidence of 1.1 (0.9–1.3) and 451 strokes among users of DPP‐4i with a cumulative incidence of 1.6 (1.5–1.8).

Table 3 shows that new users of GLP‐1 RA had a lower incidence of stroke when compared to new users of DPP‐4i, Adjusted cause‐specific hazard rate ratio (aHRR): 0.69 (0.53–0.91). There was the same signal in SGLT2i versus new users of DPP‐4i; however, this was not statistically significant (aHRR: 0.80 (0.64–1.01)). There was no difference in stroke incidence between the new users of SGLT2i versus GLP‐1 RA: aHRR 1.17 (0.64–1.01).

New users of GLP1‐RA; SGLT2i and DPP‐4i showed similar rate of myocardial infarction when compared (Table 3). Sensitivity analyses with exclusions of persons with prior myocardial infarction and TIA, respectively, were in line with the main analyses (Appendix S1).

All‐cause mortality was lower among new users of GLP‐1 RA and SGLT2i compared to new users of DPP‐4i with an aHRR of 0.44 (0.36–0.53) and 0.40 (0.34–0.48), respectively. There was no difference in mortality between SGLT2i versus GLP‐1 RA: aHRR 0.88 (0.69–1.11) (Table 3).

## 4 Discussion

In a nationwide register‐based cohort of individuals with type 2 diabetes who were new users of either GLP‐1 RA, SGLT2i or DPP‐4i and without prior stroke, the risk of stroke was lower among the users of GLP‐1 RA compared to those receiving DPP‐4i. New users of GLP‐1 RA and SGLT2i had lower all‐cause mortality when compared to new users of DPP‐4i. The risk of myocardial infarction, however, was comparable between the groups. Altogether, the results suggest that GLP‐1 RA receptor agonists were associated with significantly lower risk of stroke compared to DPP‐4i in persons with type 2 diabetes without prior stroke. There was the same signal for SGLT2i; however, this was not significant in the adjusted analysis.

Although it is well‐established that persons with type 2 diabetes are at a significantly higher risk of a stroke and that cardiovascular outcome trials have documented that GLP‐1 RA and SGLT2i reduce the risk of major cardiovascular events in clinical trial settings, stroke is often disregarded when examining new treatment strategies while information on cardiac outcomes, chronic kidney disease and heart failure is more frequently available. A recent review underlines the unmet need to reduce the burden of stroke in type 2 diabetes and highlights the importance of further research in this field [8].

One of the main findings of our study was the lower risk of stroke in new users of GLP‐1 RAs when compared to users of DPP‐4i. The same signal was seen in new users of SGLT2i compared to DPP‐4i users. However, there was no difference in the risk of stroke between GLP‐1 RA and a SGLT2i in the cohort consisting of persons with type 2 diabetes without prior stroke but possible other cardiovascular diseases. These results are in line with recent studies. One found that individuals initiating semaglutide (a GLP‐1 RA) had a lower risk of stroke with a short‐term follow‐up (median follow‐up duration 237–254 days) than those initiating a DPP4i [25]. Another recent study found both SGLT2i and GLP‐1 RAs to be associated with lower risk of major adverse cardiovascular events compared with DPP‐4i but there was no difference between GLP‐1 RA and SGLT2i [26]. The mechanisms underlying the benefit of a GLP‐1 RA or a SGLT2i on cardiovascular outcomes are not entirely clear. Putative mechanisms that may explain the beneficial properties of GLP‐1 RA and SGLT2i inhibitors include weight loss; reduction in blood pressure; off‐target pleiotropic effects related to reduced inflammation and oxidative stress; and improved vascular endothelial function [27, 28, 29].

Another main finding was the association between a lower risk of all‐cause mortality in new users of GLP‐1 RAs or a SGLT2i when compared to users of DPP‐4i. However, there was no difference in the risk of mortality between GLP‐1 RA and SGLT2i. This result is in line with several other studies, and the reduction in cardiovascular events, for example, strokes, seems to be an important mediator of the lower all‐cause mortality [30, 31].

The finding that there was no difference of myocardial infarction in the comparison of the groups in the adjusted analysis is in line with some studies showing equal risk. However other previous evidence documents GLP‐1 RA and SGLT2i are superior in protecting against myocardial infarction in comparison with DPP4i [32, 33]. To investigate the outcome myocardial infarction further, we subsequently performed a sensitivity analysis excluding persons with prior myocardial infarction from the study cohorts due to the known risk of recurrent myocardial infarction in this group being high. In the sensitivity analysis, the outcome myocardial infarction corresponded to the primary analysis, and nor did it change the results on stroke and mortality outcomes.

As patients with a prior TIA also are at a higher risk of a stroke, we also performed a sensitivity analysis excluding patients with prior TIA from the population. However, this did not change the adjusted results (Appendix S1).

A notable strength of this study was the possibility to compare several clinical outcomes in a complete contemporary national cohort of persons with type 2 diabetes without prior stroke but possibly with other prior cardiovascular diseases who were new users of either a GLP‐1 RA, SGLT2i or DPP‐4i. In addition, we had a possibility to adjust for a wide range of potential confounding factors, including detailed variables on co‐morbidity, medication and socio‐economic factors of each person. Although we did not adjust for the difference in eGFR as a measure of nephropathy/kidney function due to 30% missing values, kidney function was taken into account by adjusting for CCI that includes moderate and severe renal disease. At the moment, randomised controlled trials (RCTs) comparing the newer glucose lowering medications head‐to‐head are lacking. Although a RCT would provide level 1 evidence of the safety and efficacy of medications, participants in RCTs represent only a small proportion of potential real‐world users of the medications due to the highly selective criteria [34, 35, 36, 37]. This emphasises the importance and value of real‐world comparative effectiveness studies that describe the performance of different therapeutics in broader populations.

This study also has limitations, which should be kept in mind when interpreting the results.

Stroke was not subdivided according to type as ischemic or hemorrhagic stroke as the percentage of hemorrhagic stroke was very low (≈10% or lower, data are presented in Appendix S1) and some strokes were classified as both ischemic and hemorrhagic; hence too few and uncertainties to do meaningful analysis on the subdivided types of strokes.

Being an observational study, it is inherently impossible to verify the complete absence of residual confounding. Furthermore, some precaution is also warranted regarding the potential risk of misclassification of the exposure, that is, redeemed prescriptions were used as a proxy measure for actual medicine use, and the fact that information on all covariates was not complete for all individuals. Ultimately, RCTs comparing new users of the different classes of glucose‐lowering medication restricted to populations with type 2 diabetes would be needed to draw firm causal conclusions about GLP‐1 RA, SGLT2i and DPP‐4i effects on risk of stroke in persons with type 2 diabetes without prior stroke.

## 5 Conclusion

New users of GLP‐1 RA in persons with type 2 diabetes had a lower risk of stroke and mortality in comparison with new users of DPP‐4i. New users of SGLT2i had a lower risk of mortality compared to DPP‐4i. New users of GLP‐1 RA, SGLT2i and DPP‐4i experienced similar risk of myocardial infarction. These data could potentially help to guide choice of glucose lowering therapy in persons with type 2 diabetes.

## Author Contributions

Sidsel Hastrup; Jakob N. Hedegaard; Grethe Andersen; Merete Osler; Jørgen Rungby and Søren Paaske Johnsen all contributed to (1) conception and design, acquisition of data or analysis and interpretation of data (2) drafting the article or reviewing it critically for important intellectual content and made a (3) final approval of the version to be published.

## Funding

Funded by a research grant from Novo Nordisk A/S, Denmark.

## Consent

Under Danish law, registry‐based studies require no ethical approval or patient consent.

## Conflicts of Interest

The authors declared the funding as a potential conflicts of interest. However, Novo Nordisk A/S, Denmark had no influence on data collection, no data access and no influence on the interpretation of the results.

## Supporting information


**Data S1:** Supporting Information.

## Data Availability

According to Danish law, it is not possible to provide public access to a dataset that is based on linkage of data from nationwide public registries. Access to Danish registry data can be granted to individual researchers only upon seeking approval from the Danish Data Protection Agency.
