# Supplementary material for: A Nationwide Danish Comparative Effectiveness Study of GLP‐1 RA, SGLT2i and DPP‐4i Treatment on Risk of Stroke, Myocardial Infarction and Mortality in Type 2 Diabetes
Source: Endocrinol Diabetes Metab. 2026 Jan 24;9(1):e70165. doi: 10.1002/edm2.70165 (PMC12831120; doi:10.1002/edm2.70165)
Supplement: Supplementary file 1 — Data S1: Supporting Information. [file EDM2-9-e70165-s001.docx]

**A nationwide Danish comparative effectiveness study of GLP-1 RA, SGLT2i and DPP-4i treatment on risk of stroke, myocardial infarction and mortality in type 2 diabetes.**

**Supplementary**

1. **Identification and classification of patients with diabetes**
2. **Definition of main drug classes**
3. **List of covariates**
4. **Charlson Score Codes**
5. **Balance diagnostics in the compared groups**
6. **Percentage of ischemic stroke (IS) of all outcome strokes**
7. **Cumulative incidences of stroke, myocardial infarctions and all-cause mortality after 365 and 730 days including sensitivity analysis with exclusion of prior myocardial infarction**
8. **Sensitivity analysis with exclusion of prior TIA patients**
9. **Identification and classification of patients with diabetes**

**Our definition of diabetes was based exclusively on information available in the Danish registries. Firstly, we defined a patient as being diabetic via prescription glucose lowering medicine (ATC: A10) use with antidiabetic indication, and secondly, by classifying these patients as either type-1 or type-2 diabetic using age at first insulin or insulin analogue use (ATC: A10A) and hospital diagnosis codes (ICD-10: E10 and E11).**

**For the first part, we retrieved the entire available prescription medicine history of medicine with ATC codes beginning with A10 and filtered by**

1. **removing all prescriptions occurring up to one year after realized polycystic ovary syndrome (defined below),**
2. **removing all prescriptions occurring up to one year after realized gestational diabetes (defined below), and**
3. **removing all Liraglutid (ATC: A10BJ02) prescriptions where the associated indication for ordination was obesity.**

**If any prescriptions remained that person was diagnosed as diabetic. Secondly, we defined two criteria used to determine if a diabetic patient was T1D. For the first criterion, we found the earliest instance of insulin and insulin analogue prescription (ATC: A10) and checked whether that prescription occurred before age 30. For the second criterion, we compared the number of T1D hospital diagnoses (ICD-10: E10) with the number of T2D hospital diagnoses (ICD-10: E11), by checking if the number of E10 codes was at least greater than one and was at least as great as the number of E11 diagnoses. If either of the criteria was fulfilled, the patient was defined as T1D. The remaining diabetes patients were defined as T2D.**

**We defined a patient as having realized gestational diabetes if they had a hospital diagnosis with ICD-10 codes O24.4 or O24.9. Similarly, we defined a person as having realized polycystic ovary syndrome if had a diagnosis with ICD-10 codes E28.2. Furthermore we also defined as having PCOS if they received a combination of metformin (ATC: A10BA02) and clomifene (ATC: G03GB02), where combo-prescription was defined as receiving both within a 90 day timeframe.**

1. **Definition of main drug classes**

| **Drug** | **ATC code in database** |
| --- | --- |
| SGLT2-inhibitor | A10BX09, A10BX11, A10BX12, A10BK,  A10BD15, A10BD16, A10BD20, A10BD23, |
| GLP1 receptor agonists | A10BX04, A10BX07,  A10BX10, A10BX13,  A10BX14, A10BJ,  A10AE54, A10AE56 |
| DPP4 inhibitors | A10BH, A10BD07,  A10BD12, A10BD08, A10BD09, A10BD10, A10BD11, A10BD13, A10BD18, A10BD22, |

1. **List of covariates**

| **Variable** | **Codes** | **Notes** |
| --- | --- | --- |
| Age |  | Derived from days since birth to first use of DPP-4 inhibitors, SGLT-2i or GLP-1RA |
| Male |  | Sex assigned at birth |
| Immigrant |  | Registered as immigrant or descendant of immigrants in data available from Statistics Denmark. |
| Habitation |  | Registered as living alone in the year prior to exposure. |
| Educational level |  | Highest achieved eduational level classified using ISCED levels and aggregated into "Low" (Atmost lower Secondary Education), "Medium" (At most Short-cycle teriary education and atleast Upper secondary Education) "High" (At least a Bachelors degree or equivalent teriary education) |
| Income |  | Three year income in the was above median three year income in Denmark in the year of exposure |
| Duration  of diabetes, y |  | Time (since) between first A10 prescription and exposure |
| Charlson  Comorbidity Index | See Supplementary 4 |  |
| Atrial fibrillation | I48 |  |
| Hypertension | Hospital codes ICD-10: I10-I15 or use of medicine ATC: C02,C03, C07, C08, C09 |  |
| Diabetes with chronic complications | E10.2-E10.8, E11.2-E11.8 |  |
| Peripheral vascular disease | I70-I74 or I77 |  |
| Prior  AMI | I21-I23 |  |
| Prior  Atherosclerotic cardiovascular disease (ASCVD) | I21, I23, I24, T822A, T823, KFNA, KFNB, KFNC, KFND, KFNE, KFNF, KFNG, KFNH, KFNW, KFLF, G45, I20, I25, G45, I672, I678, I679, I691, I693, I694, I695, I696, I697, I698, I708, I61, I63, I64, I65, I66, KAAL10, KAAL11, KPAE, KPAF, KPAH, KPAN, KPAP, KPAQ, KPAW99, KPAU74, KPBE, KPBF, KPBH, KPBN, KPBP, KPBQ, KPBW, KPGH10, KPCE, KPCF, KPCH, KPCN, KPCP, KPCQ, KPCW99, KPCW20, KPCU74, KPCU82, KPCU83, KPCU84, KPGE, KPGF, KPGH, KPGN, KPGP, KPGQ, KPGW99, KPGW20, KPEE, KPEF, KPEH, KPEN, KPEP, KPEQ, KPEW, KPFE, KPFH, KPFN, KPFP, KPFQ, KPFW, KPGH20, KPGH21, KPGH22, KPGH23, KPGH30, KPGH31, KPGH40, KPGH99, KPDU74, KPDU82, KPDU83, KPDU84, KPEU74, KPEU82, KPEU83, KPEU84, KPFU74, KPFU82, KPFU83, KPFU84, KPGU74, KPGU83, KPGU84, KPGU99, KPGW, KPWG, I702, I742, I743, I744, I745, I739A, I739C, E105, E115, E145, I700, I739, I748, I749, I709, I740, I741 |  |
| Prior  PAD | I74.3 , I74.5, I70.20, I73.9, E10.5, E11.5,E13.5, E14.5, I70.21, L89, L97, L98.4, M86, R02 |  |
| Former TIA | G45 without G45.3 and G45.4 |  |
| Medical obesity | E65-E66 |  |
| HbA1c | NPU27300 |  |
| e-GFR | DNK35131; DNK35302 |  |
| LDL-cholesterol | DNK35308; NPU01568; NPU10171 |  |
| Cholesterol, total | NPU01566; NPU18412 |  |
| Biguanides | A10BA, A10BD01, A10BD02, A10BD03, A10BD05, A10BD07, A10BD08, A10BD10, A10BD11, A10BD13, A10BD14, A10BD15, A10BD16, A10BD17, A10BD18, A10BD20, A10BD22, A10BD23, A10BD25 |  |
| Sulfonylureas | A10BB, A10BD04, A10BD02, A10BD06, A10BD01, A10BC01 |  |
| Glitazones | A10BG, A10BD03, A10BD04, A10BD05, A10BD06, A10BD09, A10BD12 |  |
| Insulin and analogues | A10A |  |
| Meglitinides | A10BX02, A10BX03, A10BX08, A10BD14 |  |
| Alfa-glucosidase inhibitors | A10BF, A10BD17 |  |
| Antiplatelet drug, all | B01AC06, N02BA01, B01AC30, B01AC07 ,B01AC22, B01AC04, B01AC24, B01AC25 |  |
| Statins | C10BA, C10AA |  |
| Acetyl Salicylic Acid | B01AC06, N02BA01 |  |
| Anticoagulation (Vitamin K) | B01AA03, B01AA04 |  |
| Anticoagulation (DOAK) | B01AF02, B01AE07, B01AF01, B01AF03 |  |
| Angiotensin-converting-enzyme Inhibitors or angiotensin receptor blockers | C09A, C09B, C09C, C09D |  |
| Antihypertensive drugs, all | C02,C03, C07, C08, C09 |  |

1. **Charlson Score Codes**

| **Comorbodity** | **ICD-10 codes** |
| --- | --- |
| Myocardial infarction | I21;I22;I23 |
| Congestive heart failure | I50; I11.0; I13.0; I13.2 |
| Peripheral vascular disease | I70; I71; I72; I73; I74; I77 |
| Cerebrovascular disease | I60-I69; G45; G46 |
| Dementia | F00-F03; F05.1; G30 |
| Chronic pulmonary disease | J40-J47; J60-J67; J68.4; J70.1; J70.3; J84.1; J92.0; J96.1; J98.2; J98.3 |
| Connective tissue disease | M05; M06; M08; M09;M30;M31; M32; M33; M34; M35; M36; D86 |
| Ulcer disease | K22.1; K25-K28 |
| Mild liver disease | B18; K70.0-K70.3; K70.9; K71; K73; K74; K76.0 |
| *Diabetes mellitus** | E10.0, E10.1; E10.9 E11.0; E11.1; E11.9 |
| Hemiplegia | G81; G82 |
| Moderate/severe renal disease | I12; I13; N00-N05; N07; N11; N14; N17-N19; Q61 |
| *Diabetes mellitus with chronic complications *** | E10.2-E10.8 E11.2-E11.8 |
| Any tumor | C00-C75 |
| Leukemia | C91-C95 |
| Lymphoma | C81-C85; C88; C90; C96 |
| Moderate/severe liver disease | B15.0; B16.0; B16.2; B19.0; K70.4; K72; K76.6; I85 |
| Metastatic solid tumor | C76-C80 |
|  |  |
| * = Excluded diagnoses |  |

1. **Balance diagnostics in the compared groups**

1. **Percentage of ischemic stroke (IS) of all outcome strokes**

| Time (days) | Exposure | Comparison | Stroke  (n)  crude | IS*  (n) crude | % IS of all strokes | Stroke  (n) adjusted | IS*  (n) adjusted | % IS of all strokes |
| --- | --- | --- | --- | --- | --- | --- | --- | --- |
| 365 | DPP4 | DPP4/GLP1 | 309 | 286 | 92,56 | 182 | 176 | 96,70 |
| 365 | DPP4 | DPP4/SGLT2 | 309 | 286 | 92,56 | 184 | 179 | 97,28 |
| 365 | GLP1 | DPP4/GLP1 | 67 | 66 | 98,51 | 50 | 47 | 94,00 |
| 365 | GLP1 | GLP1/SGLT2 | 67 | 66 | 98,51 | 60 | 55 | 91,67 |
| 365 | SGLT2 | DPP4/SGLT2 | 107 | 99 | 92,52 | 89 | 82 | 92,13 |
| 365 | SGLT2 | GLP1/SGLT2 | 107 | 99 | 92,52 | 82 | 73 | 89,02 |
| 730 | DPP4 | DPP4/GLP1 | 451 | 415 | 92,02 | 249 | 243 | 97,59 |
| 730 | DPP4 | DPP4/SGLT2 | 451 | 415 | 92,02 | 258 | 256 | 99,22 |
| 730 | GLP1 | DPP4/GLP1 | 100 | 97 | 97,00 | 84 | 73 | 86,90 |
| 730 | GLP1 | GLP1/SGLT2 | 100 | 97 | 97,00 | 88 | 80 | 90,91 |
| 730 | SGLT2 | DPP4/SGLT2 | 149 | 136 | 91,28 | 128 | 115 | 89,84 |
| 730 | SGLT2 | GLP1/SGLT2 | 149 | 136 | 91,28 | 116 | 105 | 90,52 |

* IS = Ischemic stroke. Some strokes were doubly marked as IS and intracerebral hemorrhage (ICH).

1. **Cumulative incidences of stroke, myocardial infarctions and all-cause mortality after 365 and 730 days including sensitivity analysis with exclusion of prior myocardial infarction.**

| Drug class | Time window (d) | Population (n) | Strokes  (n), % 95% /*#* | Myocardial infarction n, % 95% /*#* | All-cause mortality (n), % 95CI /*#* |
| --- | --- | --- | --- | --- | --- |
| GLP-1 RA |  | 19999/19007 |  |  |  |
|  | 365 |  | 67/*55*  0.4 (0.3-0.6)/*0.4 (0.3-0.5)* | 75/*47*  0.5 (0.4-0.6)/*0.3 (0.2-0.4)* | 111/*99*  0.7 (0.6-0.9)/*0.7 (0.6-0.8)* |
|  | 730 |  | 100/*84*  0.9 (0.7-1.1)/*0.8 (0.6-1.0)* | 107/*67*  0.9 (0.8-1.2)/*0.6 (0.5-0.8)* | 166/*147*  1.5 (1.3-1.7)/*1.4 (1.2-1.7)* |
| SGLT2i |  | 24702/22676 |  |  |  |
|  | 365 |  | 107/*92*  0.6 (0.5-0.7)/*0.5 (0.4-0.7)* | 153/*55*  0.8 (0.6-0.9)/*0.3 (0.3-0.4)* | 170/*145*  0.9 (0.8-1.1)/*0.8 (0.7-1.0)* |
|  | 730 |  | 149/*129*  1.1 (0.9-1.3)/*1.0 (0.8-1.2)* | 202/*86*  1.3 (1.1-1.5)/*0.7 (0.6-0.9)* | 223/*194*  1.5 (1.3-1.7)/*1.4 (1.2-1.7)* |
| DPP-4i |  | 41943/39160 |  |  |  |
|  | 365 |  | 309/2*73*  0.9 (0.8-1.0)/*0.9 (0.8-1.0)* | 250/*158*  0.7 (0.7-0.8)/*0.5 (0.4-0.6)* | 1284/*1111*  3.8 (3.6-4.0)/*3.5 (3.3-3.7)* |
|  | 730 |  | 451/*403*  1.6 (1.5-1.8)/*1.6 (1.4-1.7)* | 358/*247*  1.3 (1.1-1.4)/*1.0 (0.9-1.1)* | 1819/*1569*  6.4 (6.1-6.7)/*5.9 (5.6-6.2)* |

# Sensitivity analyses – persons prior myocardial infarction excluded from the study populations.

|  | Population (n) | Stroke (n) | Stroke (HRR) | MI (n) | MI (HRR) | Mortality (n) | Mortality (HRR) |
| --- | --- | --- | --- | --- | --- | --- | --- |
| GLP1-RA / DPP-4i |  |  |  |  |  |  |  |
| Crude  *#* | 61,942  *58,167* | 551  *487* | 0.50 (0.40-0.62)  *0.65 (0.49-0.88)* | 465  *314* | 0.68 (0.54-0.84)  *0.61 (0.47-0.80)* | 1985  *1716* | 0.21 (0.18-0.24)  *0.21 (0.18-0.25)* |
| Adjusted*  *#* | 40,631  *38,103* | 319  *272* | 0.69 (0.53-0.91)  *0.62 (0.46-0.84)* | 290  197 | 1.00 (0.76-1.32)  *1.06 (0.75-1.50)* | 834  *724* | 0.44 (0.36-0.53)  *0.43 (0.35-0.54)* |
| SGLT2i / DPP-4i |  |  |  |  |  |  |  |
| Crude  *#* | 66,645  *61836* | 600  *532* | 0.63 (0.52-0.76)  *0.62 (0.51 - 0.76)* | 560  *333* | 1.07 (0.90-1.27)  *0.69 (0.54-0.88)* | 2042  *1763* | 0.23 (0.20-0.27)  *0.24 (0.21-0.28)* |
| Adjusted*  # | 43,810  *41333* | 370  *336* | 0.80 (0.64-1.01)  *0.80 (0.62-1.03)* | 337  *202* | 1.19 (0.94-1.49)  *0.87 (0.60-1.18)* | 1027  *906* | 0.40 (0.34-0.48)  *0.40 (0.34-0.48)* |
| SGLT2i / GLP-1 RA |  |  |  |  |  |  |  |
| Crude  # | 44,701  *41,683* | 249  *213* | 1.26 (0.97-1.62)  *1.34 (1.02-1.76)* | 309  *153* | 1.59 (1.26-2.01)  *1.12 (0.81-1.54)* | 389  *341* | 1.13 (0.92-1.38)  *1.15 (0.93-1.42)* |
| Adjusted*  *#* | 34,448  *31986* | 202  *176* | 1.17 (0.87-1.57)  *1.17 (0.86-1.60)* | 256  *129* | 1.31 (1.00-1.70)  *0.87 (0.60-1.26)* | 315  *279* | 0.88 (0.69-1.11)  *0.89 (0.69-1.14)* |

*Adjusted for age, sex, calendar year of initiation, migrant status, co-habitation status, income, education, duration of diabetes, hypertension, atrial fibrillation, Charlson Comorbidity Index (CCI), prior medication with statins, antiplatelets, anticoagulation (Vitamin K and DOAC) and diabetes-related chronic complications.

# Sensitivity analyses – prior myocardial infarction excluded from the population.

1. **Sensitivity analysis with exclusion of prior TIA patients**

**Stroke:**

**Myocardial infarction:**

**Mortality:**
